# Impact of transgenic insect-resistant maize HGK60 with *Cry1Ah* gene on community components and biodiversity of arthropods in the fields

**DOI:** 10.1371/journal.pone.0269459

**Published:** 2022-06-03

**Authors:** Yanjun Chen, Mengyun Ren, Libo Pan, Bo Liu, Xiao Guan, Jun Tao

**Affiliations:** 1 Chinese Research Academy of Environmental Sciences, Beijing, P.R. China; 2 College of Tropical Crops, Hainan University, Haikou, P.R. China; 3 Institute of Crops and Nuclear Technology Utilization, Zhejiang Academy of Agricultural Sciences, Hangzhou, P.R. China; Manonmaniam Sundaranar University, INDIA

## Abstract

In recent years, transgenic technology has developed rapidly, but the risk of the environmental release of transgenic organisms is still a key issue. Research on the impact on biodiversity is an effective way to objectively evaluate the risk. By taking transgenic maize HGK60 with insect-resistant gene *Cry1Ah* and common maize Zheng 58 as control, a 2-year experiment of arthropod community biodiversity in fields of them were studied using three methods.in 2019 and 2020. The results showed that a total of 124 species and 38537 individuals were observed from the experiment, belonging to 11 orders and 40 families. There was no significant difference in the individual number and species number of herbivorous, predatory and parasitic groups in the two kinds of maize in two years. Only the individual number of HGK60 was significantly higher than that of common maize Zheng 58 at heading stage in 2019. And the percentages of individual number and species number in different groups were basically the same in the two kinds of maize at each stage in two years. Analyses of Richness index, Shannon-Wiener diversity index, Dominance index and Evenness index showed no significant difference between the two kinds of maize in two years. The similarity coefficient of the arthropod community suggested that the arthropod community composition of HGK60 was similar to that of common maize Zheng 58. Furthermore, HGK60 had no significant effect on the relative stability of the arthropod community. These results indicated that despite the presence of a relatively minor difference in arthropod community between the two kinds of maize, the planting of HGK60 had little effect on arthropod community biodiversity. The results provided some data and support for the further studies of environmental risk of transgenic crops.

## 1. Introduction

With the ever-growing human population, food security is the major concern of this century [1]. Thus, obtaining higher crop yield per capita through various sustainable approaches has become more and more important. Genetically modified (GM) crops, which are the introduction of beneficial genes in a crop plant, can provide sustainable agronomic and economic benefits [2]. GM crops have been commercialized for 26 years, and some of them such as insect resistant, herbicide resistant, combined insect and herbicide resistant and viral disease resistant crops are being cultivated in different parts of the world [3,4]. The planting area of GM crops was 190.4 million hectares, an increase of 112 times over 1996, 2.7 billion hectares represents the total planting area from 1996 to 2019 [5]. Great progress has also been made in the research and development of GM crops in China. Transgenic cotton, poplar, sweet pepper, papaya and other crops have been released in the environment for many years, and transgenic insect-resistant rice, phytase transgenic maize and other crops also have the technical capacity of commercial production. Although global commercialization of GM crops has rapidly increased in the past 26 years, the safety of GM crops is still the most important factor restricting the further development of industry. The safety issues of GM crops mainly include food safety and environmental safety [6]. In order to solve the safety issues, the international organizations and some countries have made great efforts to improve the safety of GM organisms. There are promulgated laws and regulations on the safety management of GM organisms [7–10], which require that the research and development of GM organisms must be evaluated in advance on food safety and environmental safety. Although the safety evaluation can be used for commercial production and application, safety management measures including environmental monitoring should be taken in the process of production and application. In the existing environmental monitoring process, some agricultural concerns associated with commercial cultivation of GM crops have been reported [11]. In *Bt* crops for example, the target insect pests may develop Bt toxin resistance over time, making them more difficult to be controled in the future [12,13]. The fear is that the transgene flow from Bt crops to surrounding plant diversity, and the potential development of ‘super weed’ is one of the examples [14].

Maize is one of the most widely planted crops in the world, and insect pests are the main factors affecting its quality and yield. Compared with traditional maize, transgenic insect resistant maize can effectively reduce the number of target pests, reduce the occurrence of insect pests in the fields, reduce the dependence on chemical insecticides, and also slow down the impact of insecticides on the environment. Will it affect the biodiversity of arthropods in the fields? That still needs comprehensive safety measurement, which is also an important part of the environmental safety assessment of GM crops.

Arthropod communities are an important part of natural ecosystems and are important factors in maintaining the normal ecological functions of farmland ecosystem [15]. Arthropod communities promote the exchange of substance and energy in soil ecosystem through decomposing biological residues, changing soil physical-chemical properties and maintaining soil moisture and temperature [16,17]. Studying insects and natural predatory communities has an important guiding role in the practice of farmland pest control. In addition, the diversity of arthropod communities (including insects) reflects the relationship between organisms and the environment. Therefore, the diversity of arthropods in the field is an important component of the environmental safety assessment of GM crops.

Insect-resistant gene *Cry1Ah* is environmental safe product thanks to its higher toxic to a variety of lepidopterous insects and low toxicity to economic insects such as *B*ombyx mori. But, the expression of *Cry1Ah* might lead to alter plant root metabolites composition to induce changes in ambient soil biodiversity [18]. El-Wakeil and Volkmar [19] thought the Bt-transgenic maize cultivation showed no adverse effects on the biodiversity of epigeal arthropods and soil macro-organisms in the field. Some studies have proved that the fungi and their metabolites in the soil can control the insect pests [20]. Large use of chemical reagents in arthropod control resulted in pesticides resistance [21]. Insect-resistant GM crop production can improve the biodiversity of animals and plants, the biological control of pests and diseases, as well as reduction of chemical inputs [19]. It is foreseeable that microbial-based pest control could be an attractive alternative to chemical insecticides in the future. In this study, we investigated the species and quantity of arthropods in the fields and analyzed the effects of transgenic insect-resistant maize HGK60 with *Cry1Ah* gene on the diversity of arthropods, which can provide a scientific basis for the evaluation of the environmental safety of this type of GM maize.

## 2. Materials and methods

### 2.1 Experimental material and experimental design

The experimental site of this study is Langfang Agricultural High-Tech Industrial Park belongs to Chinese Academy of Agricultural Sciences(39°36’N, 116°36’E), which has a temperate semi-humid continental climate with an annual average temperature of 11.9°C, 2660 annual sunshine hours, annual average precipitation of 554.9 mm, and sandy loamand clay loam soils with neutral pH. The experimental site is being cultivated with HGK60 maize for more than 10 years.

A randomized block design was used in this study. We used the transgenic insect-resistant maize HGK60 with *Cry1Ah* gene (HGK60 for short) and the common maize Zheng 58 (Z58 for short), which were provided by Chinese Academy of Agricultureal Sciences (Beijing, China). There were 4 replicates of samples, with 10 m ×10 m per plot. 1 m wide isolation belt was set among different plots, and the planting mode was one hole one grain, one hole two grains cycle sowing. The distance between maize plants was 25 cm, and the row spacing was 60 cm. The growing season for maize is between May and September in 2019 and 2020. The maize was planted on 1 May and harvested on 1 September. No fertilizer was applied, and no pesticides were sprayed during the growth process. Maize was bagged and artificially pollinated at heading stage. The frequency of watering was a regular half a month, topdressing period is nine leaves to eleven leaves, the weeding effect applied after the seedling and pollination.

### 2.2 Sample collection and processing

The direct observation method, trap survey method and sweeping method were used to collect samples at seedling, bell, heading and full ripe stage of the maize growth period. Each plot was sampled by a five-point sampling method three times in each growth period. The direct observation method mainly investigated the species and quantity of arthropods on plants, and 15 maize plants were investigated and recorded at each point. The trap survey method mainly investigated the species and quantity of surface arthropods. Each plot was sampled by a five-point sampling method. Each point had 5 plastic cups with a diameter of 7.5 cm, each plastic cup has a interval of 0.5 m, and a 5% antifreeze fluid is placed in the cups accounting for 1/3 of the volume of the cup. After 24 h, all the specimens were put into bottles containing 70% alcohol. The specimens were brought back to the laboratory and were screened for counting and species identification. The sampling time of sweeping method was 16:00 to 19:00 [22]. Each plot was sampled by a five-point sampling method, and 10 plants were inspected randomly at each point, with 50 samples per plot. The sampling method was carried out 10 times on the stems and leaves of the maize plant from top to bottom using an insect net with a diameter of 30 cm. All the specimens were put into bottles containing 70% alcohol and were killed. The specimens were brought back to the laboratory and were screened for counting and species identification [23]. The specimens were stored in Chinese Research Academy of Environmental Sciences. The investigation was conducted for two consecutive years.

According to the method of Hao, arthropod communities were roughly divided into four groups according to the position (trophic level) in the food chain: predatory, herbivorous, parasitic and neutral (including saprophytic groups and groups that are neither harmful to plants nor eat other insects). Species in Araneida (Arachnida) were classified as predatory groups [24].

### 2.3 Data analysis

A 2-year experiment of community composition and abundances of arthropods in fields was carried out. The community structure and composition of arthropods in the fields were analyzed. Survey data were presented as the means ± standard deviation of 4 replicates. Statistical analysis was performed by t-test using SPSS 16.0. A significance level of 0.05 was set for all tests.

Richness index(Dmg): Dmg = (*S*-1)/ln*N*. Here, *S* is the number of species, *N* is the number of individuals

Diversity index(*H*):$H=\text{-}\sum_{i=\text{1}}^{\text{s}}Pi\ln Pi$. Here, *Pi* is the ratio of the number of the *i*th individual to the total number of individuals.
Dominance index(*D*):$D=1\text{-}\sum_{i=1}^{\text{s}}Pi^{2}$
Evenness index(*J*): *J* = *H*/ln*S* [25–27]

The community similarity coefficient used was the Sorensen or Zekanovski coefficient (*C’*): *C’ =* 2*W*/(a+b) [28], where *W* is the number of species shared by HGK60 and Z58, a is the total number of species in HGK60, and b is the total number of species in Z58. Values of 0.75 ≤ *C’* < 1.0 indicate very similar, 0.5 ≤ *C’* < 0.75 indicates moderately similar, 0.25 ≤ *C’* < 0.5 indicates moderately dissimilar, and 0 < *C’* < 0.25 indicates very dissimilar.

The regulation of natural group and neutral group on herbivorous groups in arthropod community was reflected by the numerical value of *N*n/*N*p, *N*d/*N*p, *S*n/*S*p and *S*d/*S*p. *N*n/*N*p is the ratio of the number of individuals of natural group to that of herbivorous group; *N*d/*N*p is the ratio of the number of individuals of neutral group to that of herbivorous group; *S*n/*S*p is the ratio of the number of species of natural group to that of herbivorous group; *S*d/*S*p is the ratio of the number of species of neutral group to that of herbivorous group [29]. The species of natural group in this study included both predatory and parasitic groups.

## 3. Results

### 3.1 Arthropod community composition in the fields

A total of 124 species and 38537 individuals were observed from the experiment, belonging to 11 orders and 40 families (S1 Table). Among them, there were 4 families (10.00%) and 12 species (9.68%) of Lepidoptera, 6 families (15.00%) and 19 species (15.32%) of Hemiptera, 4 families (10.00%) and 22 species (17.74%) of Homoptera, 3 families (7.50%) and 9 species (7.26%) of Orthoptera, 1 family (2.50%) and 4 species (3.23%) of Neuroptera, 3 families (7.50%) and 12 species (9.68%) of Coleoptera, 1 family (2.50%) and 2 species (1.61%) of Thysanoptera, 6 family (15.00%) and 16 species (12.90%) of Diptera, 8 family (20.00%) and 14 species (11.29%) of Hymenoptera, 1 family (2.50%) and 2 species (1.61%) of Odonata, and 3 family (7.50%) and 12 species (9.68%) of Araneae (Table 1).

**Table 1:** Community composition of arthropods in the maize fields.

Orders	Families	Percentage %	Species	Percentage %
Lepidoptera	4	10.00	12	9.68
Hemiptera	6	15.00	19	15.32
Homoptera	4	10.00	22	17.74
Orthoptera	3	7.50	9	7.26
Neuroptera	1	2.50	4	3.23
Coleoptera	3	7.50	12	9.68
Thysanoptera	1	2.50	2	1.61
Diptera	6	15.00	16	12.90
Hymenoptera	8	20.00	14	11.29
Odonata	1	2.50	2	1.61
Araneae	3	7.50	12	9.68
Total	40	-	124	-

Comparing the number of arthropods in the field for the same growth period, the number of Lepidoptera arthropods of HGK60 was still higher than that of Z58 in each growth, and the number of other arthropods was similarly between HGK60 and Z58 (S2 Table).

**Fig 1:**
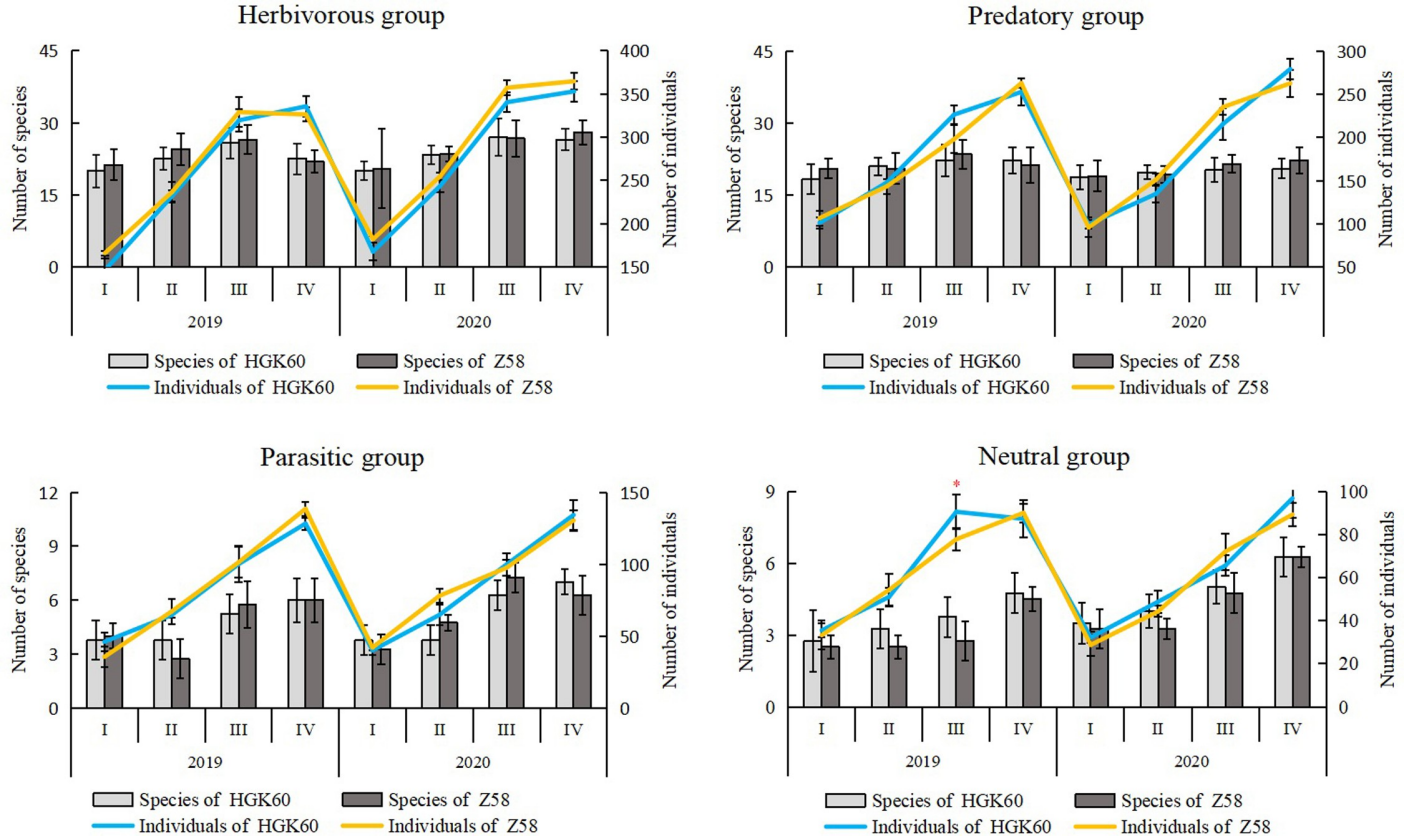
Species number and individual number of the herbivorous, predatory, parasitic and neutral groups of arthropods in the fields of HGK60 and Z58 in 2019 and 2020. I: Seedling stage; II: Bell stage; III: Heading stage; IV: Full ripe stage. Data (means ± SD) in the figure followed by asterisks indicate a significant difference betweenHGK60 and Z58 by t-test at *P* < 0.05. The same below.

The arthropod community was statistically analyzed and classified according to feeding habits, as there were different types of feeding habits within the same arthropod taxonomic unit. The number of species and individuals of arthropods with different feeding habits on HGK60 and Z58 are shown in Fig 1. The comparison results of the phytophagous group, predatory group and parasitic group revealed no significant difference between the two kinds of maize in the number of species and individuals in each growth period of two years. And the number of individuals of them increased with the growth period. For the neutral groups, there was no significant difference between the two kinds of maize in the number of species in each growth period. But the number of individuals of HGK60 was significantly higher than that of Z58 at heading stage in 2019.

**Fig 2:**
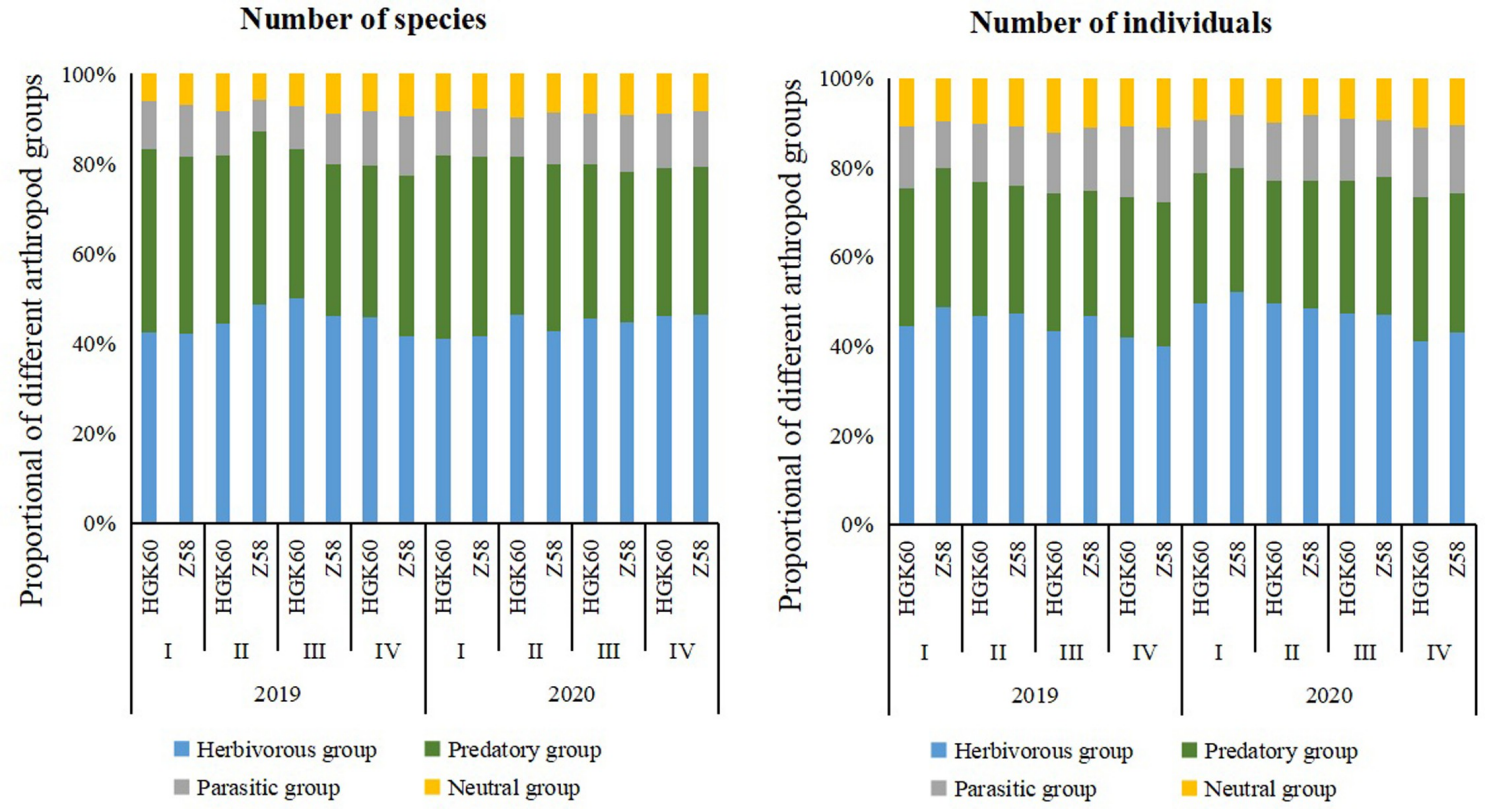
The percentage of the species number and individual number of arthropods on HGK60 and Z58 in 2019 and 2020.

Further results showed that the species number in the herbivorous group accounted for the largest proportion of the arthropod community in the fields of both kinds of maize. In contrast, the neutral group comprised the smallest number of species during the two sampling years. The results showed that the number of individuals in each group was: herbivorous group > predatory group > parasitic group > neutral group. On the other hand, the percentages of individual number and species number in four groups were basically the same in the two kinds of maize at each stage in 2019 and 2020 (Fig 2).

### 3.2 Impacts of maize type on diversity indexes of the arthropod community

**Fig 3:**
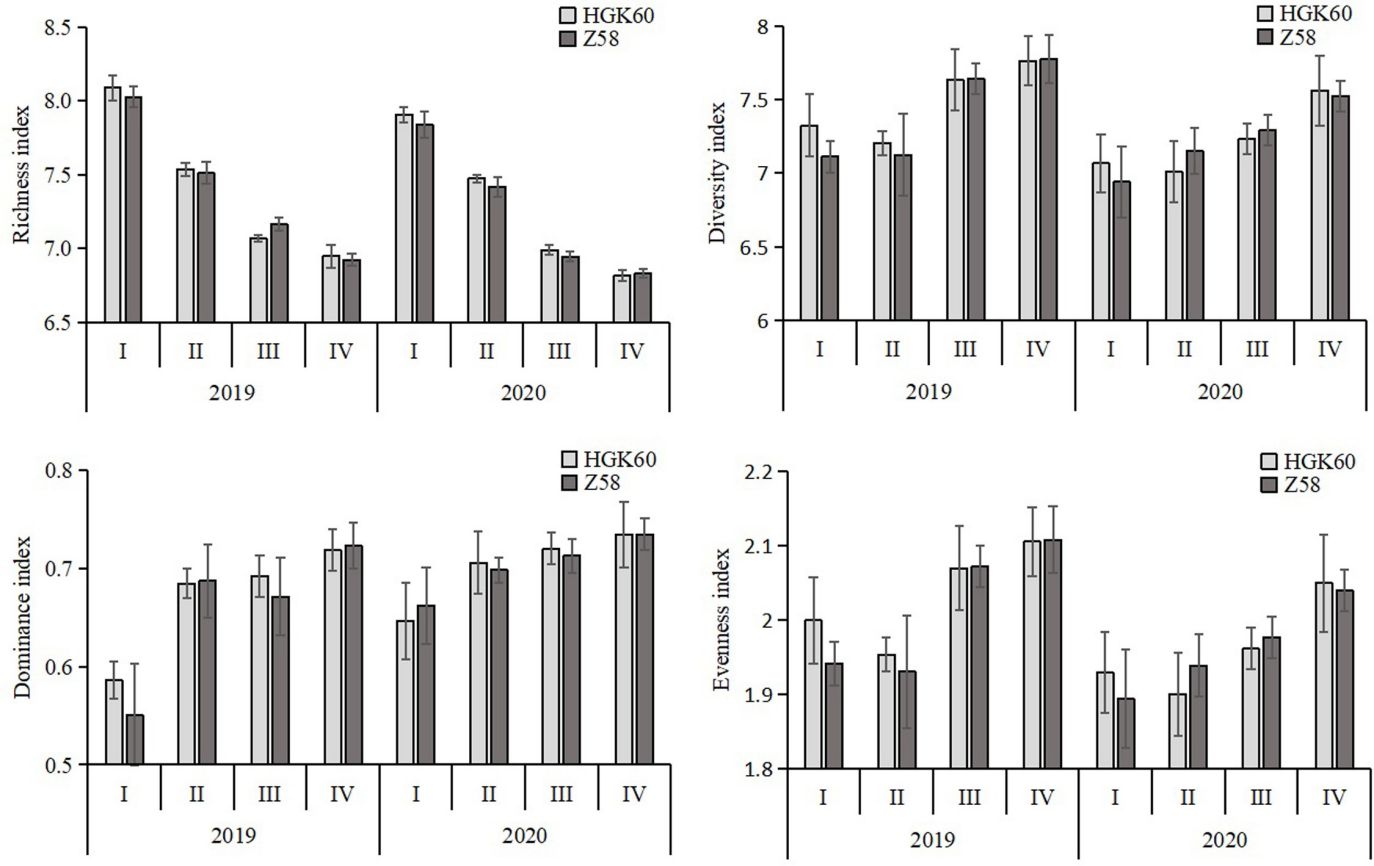
The biodiversity indexes of arthropods in the fields.

The Richness index (*D*_*mg*_), Shannon-Wiener diversity index (*H*), Dominance index (*D*) and Evenness index (*J*) were used to determine the diversity characteristics of the arthropod communities in the HGK60 and Z58 maize plots (Fig 3). Comparing the richness index of arthropod community between HGK60 and Z58, the result showed no significant difference (*P*> 0.05) between HGK60 and Z58 in the 2-year experiment. The Shannon-Wiener diversity index (H) and Dominance index (*D*) of arthropod communities in different plots showed that there was no significant difference between HGK60 and Z58 in each growth period in the 2-year experiment. The Evenness index (*J*) of arthropod communities in different plots showed similar values, ranging from 1.89 to 2.11, that in the HGK60 and Z58 maize plots showed no significant difference *(P*>0.05) in 2019 and 2020. That is, analyses of Richness index (*D*_*mg*_), Shannon-Wiener diversity index (*H*), Dominance index (*D*) and Evenness index (*J*) showed no significant difference between HGK60 and Z58 in 2019 and 2020.

The results showed that the similarity coefficients of each group (herbivorous, predatory, parasitic and neutral group) in the HGK60 and Z58 maize plots were higher than 0.6 in 2019 and 2020 (Table 2). In the two years, the similarity coefficients of arthropod communities in the HGK60 and Z58 maize plots were higher than 0.72, and the similarity coefficients at heading and full ripe stage were higher than 0.75, indicating that the arthropod communities in these plots were highly similar. According to the experiment results in 2019, the similarity coefficients of herbivorous group at the seeding stage and bell stage reached a moderately similar level, and that at heading and full ripe stage reached a highly similar level. The coefficients of predatory group was highly similar at all four growth stages. The coefficients of parasitic group was moderately similar at seedling stage and heading stage, and highly similar at bell stage and full ripe stage. The coefficients of neutral group was highly similar only at full ripe stage, and that was moderately similar at the other three growth stages. According to the experiment results in 2020, the similarity coefficients of herbivorous group at the seeding stage, heading stage and bell stage reached a highly similar level, and that at bell stage reached a moderately similar level. The coefficients of predatory group were highly similar at all four growth stages. For parasitic group and neutral group, the coefficients were slightly lower, that only reached highly similar level at full ripe stage.

**Table 2:** The similarity coefficient of the arthropod community between two maize fields (HGK60 vs. Z58).

Year	Growth period	Arthropod community	Herbivorous group	Predatory group	Parasitic group	Neutral group
2019	Seeding stage	0.7299	0.7241	0.7636	0.6667	0.6667
	Bell stage	0.7465	0.7273	0.7778	0.8333	0.6000
	Heading stage	0.7514	0.7711	0.7586	0.6667	0.7143
	Full ripe stage	0.7784	0.7945	0.7586	0.7619	0.8000
2020	Seeding stage	0.7460	0.7692	0.7843	0.6154	0.6000
	Bell stage	0.7376	0.7302	0.7843	0.7143	0.6154
	Heading stage	0.7590	0.8000	0.7500	0.7000	0.6667
	Full ripe stage	0.7979	0.7586	0.8387	0.7826	0.8750

It can be seen from Table 3 that in the 2-year experiment, the stability of arthropod community in HGK60 and Z58 maize fields showed little difference in the same growth period. The difference of *N*d/*N*p between HGK60 and Z58 was significant only in the seedling stage of 2020, while the difference of *N*n/*N*p, *S*n/*S*p and *S*d/*S*p were not significant in each growth period in the 2 years. In general, the stability indexes of arthropod community of HGK60 and Z58 were relatively stable, that indicated that the planting of HGK60 did not interfere with the regulation of natural group and neutral group on herbivorous group in arthropod community.

**Table 3:** Comparison of stability of arthropod community in maize fields.

Year	Growth period	Maize treatments	Stability index				
			*N*_n_/*N*_p_	*N*_d_/*N*_p_		*S*_n_/*S*_p_	*S*_d_/*S*_p_
2019	Seeding stage	HGK60	1.021±0.141	0.244±0.032		1.133±0.253	0.152±0.104
		Z58	0.857±0.033	0.201±0.05		1.182±0.273	0.117±0.015
	Bell stage	HGK60	0.917±0.018	0.22±0.02		1.112±0.181	0.147±0.045
		Z58	0.892±0.076	0.231±0.049		0.993±0.379	0.107±0.041
	Heading stage	HGK60	1.022±0.048	0.283±0.021		1.086±0.232	0.144±0.019
		Z58	0.915±0.12	0.237±0.016		1.122±0.215	0.107±0.047
	Full ripe stage	HGK60	1.136±0.063	0.26±0.029		1.289±0.288	0.218±0.065
		Z58	1.232±0.05	0.276±0.016		1.244±0.11	0.205±0.022
2020	Seeding stage	HGK60	0.835±0.072	0.192±0.015	*	1.142±0.258	0.174±0.038
		Z58	0.759±0.075	0.159±0.037		1.117±0.373	0.161±0.051
	Bell stage	HGK60	0.821±0.092	0.201±0.03		1.024±0.206	0.172±0.033
		Z58	0.899±0.059	0.174±0.014		1.02±0.054	0.139±0.021
	Heading stage	HGK60	0.927±0.058	0.192±0.012		1.016±0.292	0.186±0.017
		Z58	0.931±0.014	0.201±0.025		1.091±0.144	0.184±0.061
	Full ripe stage	HGK60	1.172±0.045	0.274±0.032		1.047±0.167	0.237±0.039
		Z58	1.078±0.04	0.245±0.018		1.025±0.12	0.226±0.036

*N*_n、_*N*_p、_*N*_d、_*S*_n、_*S*_p_ and *S*_d_ in the table refer to the number of individuals of natural group, the number of individuals of herbivorous group, the number of individuals of neutral group, the number of species of natural group, the number of species of herbivorous group, the number of species of neutral group.

## 4. Discussion

In this study, the results showed that there was no significant difference on the composition, diversity indexes, similarity coefficient and relative stability of the arthropod community between transgenic insect-resistant maize (HGK60) and the non-GM maize. Previous studies have found that the impact of transgenic crops on soil fauna, resulting in minimal to no effects on biodiversity [4,18,30–32]. Guo et al. [33] suggested that transgenic Bacillus thuringiensis (*Bt*) maize had little effect on natural enemy community biodiversity. It is well known that transgenic insect-resistant maize can specifically kill target insects, so it is common to study its nontarget effects on the biodiversity of natural enemy communities [22]. Wang et al. [22] investigated the composition and abundance of arthropod community in transgenic maize fields for two years in Beijing. As for the number of species, individuals, community diversity indexes, similarity coefficient and relative stability of arthropod community, the results showed that there was no significant difference between transgenic insect-resistant maize and the non-GM maize. Through the observation and statistics of the influence on the biodiversity of arthropod in the field in Changchun, Yin et al. [34] found that there was no significant difference between the community composition, community structure and the control maize of Bt-799, and the seasonal variation was also consistent. Guo et al. [35] used four methods of direct observation, air basin trap, ground trap and insect attractor for 2 years to obtain that the number of arthropods, diversity index, dominance concentration index in Langfang, and evenness index of arthropod in fields of transgenic maize are not significantly different from those of the control, which indicates that the planting of transgenic maize with *Cry1Ie* gene had no significant effect on the structure and diversity of arthropod community in the fields. Habustova et al. [36] had been monitoring the arthropod in the fields of transgenic Bt maize for 3 years in a row in ČESKÉ BUDĚJOVICE, and it is found that there is no significant difference in biomass and species abundance between the transgenic Bt maize and the control. In addition, an ecological risk assessment of GM soybean also showed that the cultivation of GM soybean had no effects on the arthropod damage [37]. Similarly, Bt cotton and transgenic Cry1Ab rice has no adverse effects on *Chrysoperla carnea* in terms of development, survival, fecundity, or population dynamics [38] and the development, mummy weight and the number of progeny produced by *Copidosoma floridanum* [39] and the survival, development time and fecundity of *Pardosa pseudoannulata* in the laboratory or on predation under field conditions [40]. These studies indicated that some GM crops have no adverse effects on arthropods, which is consistent with our research. However, some scholars believe that the planting of transgenic crops will have a certain impact on the composition and organization of ecological communities. Jiang et al. [41] found that the composite system of transgenic poplar and cotton formed by transgenic cotton and 741 transgenic cotton had some influence on the composition and structure of insect sub community in the system. Some studies suggested that the planting of transgenic insect-resistant crops significantly reduced the use of insecticides, caused the increase of abundance and diversity of arthropod community in the fields, and then the ecological stability of the planting area was improved [42,43]. Meanwhile some studies showed that the planting of transgenic insect-resistant crops could cause the decrease of the number of natural enemies and the increase of the number of non-target insects in the fields to some extents [44,45]. It is still controversial that whether transgenic crops can affect arthropods in the fields. Although this study thought that HGK60 planting has no obvious influence on the composition, diversity indexes, similarity coefficient and relative stability of the arthropod community in the fields, field biosafety evaluation is a long-term research process, which needs to be tested in multiple locations for many years to enrich the repeated test results, for getting a more comprehensive and reliable conclusion. It should be noticed that there was a limitation of the study that we didn´t sample the arthropods in the different soil profiles. This will be our research direction in the future.

The research on the impact of biodiversity is a necessary link for the safe release of genetically modified crops in the environment. At present, experts and scholars have done a lot of research on the rhizosphere soil microorganisms [46–51], insects [34,36,41], weeds [14] of genetically modified crops. However, the conclusions are not the same. The differences of research results indicate that the effects of genetically modified crops on biodiversity were not the same. The differences of receptor types, exogenous genes, selection evaluation indexes and research methods may affect the research results. Therefore, the safety evaluation of genetically modified crops should follow the "case by case principle" [52]. From the ecological security perspective of GM maize, the planting scale and time are relatively limited, and the direct and indirect ecological risks are still unclear. Therefore, it is necessary to conduct long-term multi-site and multi-level systematic monitoring to ensure the safety of environmental ecosystems containing transgenic maize.

## 5. Conclusion

In this study, the planting of HGK60 had no significant influence on the composition, diversity indexes, similarity coefficient and relative stability of the arthropod community in the fields during 2-year experiment. The difference only occurs in individual growth period, and does not continue to appear in the whole growth period, such as the number of individuals in the neutral groups of HGK60 was significantly higher than that of Z58 only at heading stage in 2019, while the difference of Nd/Np between HGK60 and Z58 was significant only at the seedling stage in 2020. Furthermore, biosafety assessment for field crops is a long-term research process that requires long-term monitoring of GMOs approved for large-scale release to obtain more comprehensive evaluation results.

## Supporting information

S1 Table Patterns of the arthropod community in the fields in different orders, families and species. (DOCX)

S2 Table The number of arthropods in each growth period (heads). (DOCX)

S1 Raw data (XLSX)
