# Supplementary material for: Impact of transgenic insect-resistant maize HGK60 with Cry1Ah gene on community components and biodiversity of arthropods in the fields
Source: PLoS One. 2022 Jun 3;17(6):e0269459. doi: 10.1371/journal.pone.0269459 (PMC9165892; doi:10.1371/journal.pone.0269459)
Supplement: S1 Table — (DOCX) [file pone.0269459.s001.docx]

Table S1 Patterns of the arthropod community in the fields in different orders, families and species

| **Orders** | **Families** | **Species** |
| --- | --- | --- |
| Lepidoptera | Pyralidae | *Pyrausta nubilalis、Chilo suppressalis、Scirpophaga incertulas、Catagela adjurella、Conogethes punctiferalis、Pyralidae、Cnaphalocrocis medinalis、Chilo infuscatellus* |
|  | Hesperiidae | *Ampittia dioscorides* |
|  | Pieridae | *Pierisrapae、Aporia crataegi* |
|  | Pterophoridae | *Pterophorus monodactylus* |
| Hemiptera | Miridae | *Apolygus lucorum、Cyrtorrhinus chinesis、Tenuis、Adelphocoris lineolatus、Adelphocoris fsdciaticollis* |
|  | Nabidae | *Nabis sinoferus、Gorpis brevilineatus* |
|  | Lygaeidae | *Lygaeus equestris、Tropidothorax elegans Distant、Geocoris pallidipennis* |
|  | Anthocoridae | *Orius similis、Orius sauteri、Orius minutus* |
|  | Reduviidae | *Coranus lativentris、Haematoloecha nigrorufa、Oncocephalus confusus* |
|  | Coreidae | *Riptortus pedestris、Cores marginatus、Ochrochira camelina* |
| Homoptera | Delphacidae | *Sogatella furcifera、Saccharosydne procerus、Terthron albovittata、Nilaparvata lugens、Nilaparavata bakeri、Nilaparvata muiri、Laodelphax striatellus* |
|  | Cicadellidae | *Cicadella viridis、Nephotettix bipunctatus、Inazuma dorsalis、Thaia rubiginosa、Erythroneura sudra、Macrosteles quadrimaculata、Cotton leafhopper、Jacobiasca formosana、Tettigoniella spectra* |
|  | Aphidoidea | *Rhopalosiphum maidis、Sitobion avenae、Myzus persicae、Lipaphis erysimi pseudobrassicae* |
|  | Psyllidae | *Psyllia chinensis、Anomoneura mori Schwarz* |
| Orthoptera | Gryllidae | *Teleogryllus emma、Loxoblemmus doenitzi Stein* |
|  | Acrididae | *Oedaleus decorus asiaticus、Patanga japonica、Acrida cinerea、Locusta migratoria manilensis* |
|  | Tetrigoidea | *Aiolopus tamulus、Tetrigid、Eucriotettix oculatus* |
| Neuroptera | Chrysopidae | *Chrysoperla sinica、Chrysopa pallens、Chrysopa formosa Brauer、Chrysopa intima* |
| Coleoptera | Carabidae | *Chlaenius micans Fabricius、Calosoma Chinese、Chlaenius Posticalis、Scarites terricola、Harpalus griseus* |
|  | Coccinellidae | *Propylaea japonica、Harmonia axyridis、Adalia bipunctata、Henosepilachna pusillanima、Coccinella septempunctata、Adonia variegata* |
|  | Staphylinidae | *Paederus fuscipes* |
| Thysanoptera | Thripoidea | *Frankiniella tenuicornis、Franjliniella occidentalis* |
| *Diptera* | Syrphidae | *Dideopsis aegrotus、Eristalis tenax、Sphaerophoria sp.、Bacch maculata* |
|  | Tephritidae | *Bactrocera cucurbitae、Bactrocera Minax* |
|  | Tachinidae | *Exorista civilis、Lydella grisescens、Cuphocera varia* |
|  | Muscidae | *Musca domestica、Muscina stabulans、Stomoxys calcitrans、Fannia canicularis* |
|  | Sarcophagidae | *Parasarcophaga crassipalpis* |
|  | Drosophilidae | *Drosophila melanogaster、Drosophila pseudoobscura* |
| Odonata | Coenagrionidae | *Ischnura asiatica、Ischnura elegans* |
| Hymenoptera | Ichneumonidae | *Charops bicolor、Vulgichneumon leucaniae、Campoletis chlorideae、Coccygomimus disparis、Diplozon laetatorius、Butenyacra picta* |
|  | Trichogrammatidae | *Trichogramma ostriniae* |
|  | Encyrtidae | *Aphidencyrtus aphidivorus* |
|  | Chrysididae | *Chrysis shanghaiensis* |
|  | Sphecidae | *Sceliphron deforme、Sphex umbrosus* |
|  | Pteromalidae | *Dibrachys xavus* |
|  | Mymaridae | *Lymaenon sp.* |
|  | Braconidae | *Macrocentrus cingulum* |
| Araneae | Araneidae | *Araneus ventricosus、Araneus ejusmodi、Araneus yuanminensis、Cyclosa argenteoalba、Cyclosa octotuberculata、Neoscone doenitzi、Neoscona theisi* |
|  | Thomisidae | *Misumenops tricuspidatus、Xysticus ephippiafus* |
|  | Salticidae | *Marpissa elongata、Myrmarachne formicaria、Sitticus sinensis* |
