# Supplementary material for: Impact of transgenic insect-resistant maize HGK60 with Cry1Ah gene on community components and biodiversity of arthropods in the fields
Source: PLoS One. 2022 Jun 3;17(6):e0269459. doi: 10.1371/journal.pone.0269459 (PMC9165892; doi:10.1371/journal.pone.0269459)
Supplement: S2 Table — (DOCX) [file pone.0269459.s002.docx]

Table S2 The number of arthropods in each growth period (heads)

| Order | Family | Maize treatments | 2019 | | | | 2020 | | | |
| --- | --- | --- | --- | --- | --- | --- | --- | --- | --- | --- |
|  |  |  | Seedling  stage | Bell  stage | Heading  stage | Full ripe  stage | Seedling  stage | Bell  stage | Heading stage | Full ripe stage |
| Lepidoptera | Pyralidae | HGK60 | 7.00±1.41 | 25.75±3.70 | 30.00±3.94 | 35.25±3.83 | 10.75±1.79 | 26.00±3.39 | 39.25±2.59 | 40.25±3.27 |
|  |  | Z58 | 11.50±2.87 | 29.25±2.68 | 32.75±2.38 | 36.75±2.17 | 12.75±1.48 | 28.50±1.80 | 41.25±2.86 | 41.00±2.92 |
|  | Hesperiidae | HGK60 | 6.25±1.92 | 18±2.92 | 20.25±2.95 | 22.00±2.55 | 10.00±0.71 | 20.50±2.06 | 24.50±3.35 | 23.00±3.54 |
|  |  | Z58 | 8.25±1.92 | 18.75±3.11 | 22.25±2.17 | 23.00±1.41 | 11.25±1.48 | 22.00±4.95 | 27.25±1.92 | 23.00±1.41 |
|  | Pieridae | HGK60 | 6.25±1.63 | 10.25±1.79 | 15.50±2.96 | 18.50±2.50 | 6.00±1.87 | 9.75±2.17 | 20.25±2.95 | 21.75±2.59 |
|  |  | Z58 | 6.50±1.65 | 11.00±2.92 | 13.50±3.20 | 17.75±1.79 | 7.75±2.38 | 10.75±1.48 | 22.00±3.94 | 22.50±2.18 |
|  | Pterophoridae | HGK60 | 5.25±1.08 | 7.25±1.48 | 10.00±1.58 | 11.50±2.06 | 6.00±0.71 | 8.25±1.64 | 11.75±2.17 | 12.25±1.48 |
|  |  | Z58 | 5.25±1.78 | 7.75±1.48 | 10.25±1.64 | 11.75±2.17 | 7.25±1.48 | 9.00±1.58 | 12.75±2.68 | 12.50±1.50 |
| Hemiptera | Miridae | HGK60 | 10.00±1.41 | 11±2.55 | 19.50±3.20 | 21.25±2.86 | 10.25±1.64 | 13.25±1.30 | 22.25±2.17 | 21.25±1.92 |
|  |  | Z58 | 8.50±2.59 | 9.50±1.80 | 8.50±2.60 | 21.25±2.28 | 9.75±1.79 | 13.00±1.87 | 22.50±3.35 | 21.75±2.49 |
|  | Nabidae | HGK60 | 6.25±1.29 | 10±2.92 | 14.25±2.86 | 16.25±1.79 | 7.50±1.12 | 10.75±1.30 | 19.75±1.48 | 21.75±2.38 |
|  |  | Z58 | 6.25±2.16 | 8.25±2.28 | 6.25±2.17 | 17.25±2.59 | 7.25±1.30 | 11.00±1.22 | 21.75±3.03 | 21.25±2.28 |
|  | Lygaeidae | HGK60 | 4.25±1.47 | 6.25±1.09 | 10.75±1.92 | 12.00±1.41 | 7.00±1.58 | 8.25±1.79 | 12.50±1.80 | 14.00±2.45 |
|  |  | Z58 | 4.25±1.08 | 6.25±2.17 | 10.75±1.09 | 13.00±1.41 | 6.50±1.80 | 8.25±1.30 | 12.00±1.22 | 14.50±2.96 |
|  | Anthocoridae | HGK60 | 6.25±1.92 | 9.00±1.41 | 12.50±2.29 | 14.50±2.29 | 8.25±1.30 | 8.25±0.83 | 11.75±2.78 | 16.75±3.03 |
|  |  | Z58 | 8.25±1.92 | 8.75±1.48 | 11.50±1.66 | 14.50±1.66 | 8.75±2.28 | 9.50±1.50 | 13.25±1.79 | 16.00±3.74 |
|  | Reduviidae | HGK60 | 2.50±1.11 | 7.00±1.58 | 10.50±1.66 | 13.00±1.73 | 2.50±1.12 | 7.75±2.38 | 9.00±2.55 | 13.00±1.73 |
|  |  | Z58 | 3.25±1.47 | 6.00±1.22 | 9.00±2.74 | 12.50±2.29 | 3.25±1.48 | 7.50±2.29 | 9.75±2.28 | 12.50±2.29 |
|  | Coreidae | HGK60 | 10.00±2.12 | 12.25±0.83 | 15.75±1.92 | 20.50±1.12 | 9.00±1.22 | 11.25±1.48 | 13.50±2.96 | 20.50±1.12 |
|  |  | Z58 | 9.25±1.78 | 12.50±2.29 | 16.00±1.87 | 20.50±3.35 | 8.50±2.29 | 10.75±2.38 | 14.00±1.87 | 20.50±3.35 |
| Diptera | Drosophilidae | HGK60 | 7.75±1.29 | 10.00±1.22 | 14.50±2.29 | 15.75±1.48 | 7.75±1.30 | 9.25±1.79 | 12.00±2.55 | 15.25±2.17 |
|  |  | Z58 | 6.50±1.11 | 10.00±1.87 | 13.50±2.29 | 16.00±2.12 | 6.50±1.12 | 8.50±1.12 | 11.00±1.58 | 13.25±3.63 |
|  | Tephritidae | HGK60 | 9.00±1.58 | 11.25±2.59 | 14.25±2.59 | 17.75±2.49 | 7.75±1.92 | 9.25±1.92 | 13.25±2.86 | 17.75±2.49 |
|  |  | Z58 | 8.00±0.70 | 11.25±2.86 | 14.25±3.11 | 17.75±2.28 | 7.25±0.83 | 10.25±1.92 | 15.25±3.03 | 17.75±2.28 |
|  | Tachinidae | HGK60 | 7.50±1.65 | 10.00±1.58 | 13.00±1.87 | 16.75±1.92 | 7.00±1.58 | 9.75±2.28 | 12.25±2.86 | 20.75±3.27 |
|  |  | Z58 | 5.50±2.29 | 10.25±1.92 | 13.50±3.77 | 17.00±2.92 | 7.25±1.48 | 11.50±2.18 | 13.75±2.38 | 18.25±3.27 |
|  | Muscidae | HGK60 | 4.50±2.18 | 8.00±2.92 | 16.75±2.38 | 14.50±1.66 | 4.75±0.83 | 9.75±1.92 | 12.75±1.30 | 17.50±2.18 |
|  |  | Z58 | 3.75±1.47 | 7.25±2.59 | 15.00±3.16 | 15.00±1.87 | 4.50±1.12 | 8.50±1.80 | 11.25±2.05 | 16.25±3.27 |
|  | Syrphidae | HGK60 | 23.25±2.58 | 22.50±2.96 | 38.50±3.35 | 34.25±4.60 | 19.75±2.49 | 20.25±2.59 | 25.75±3.11 | 42.00±4.64 |
|  |  | Z58 | 23.00±4.84 | 24.75±3.90 | 31.25±5.02 | 35.50±3.57 | 17.75±2.95 | 19.50±3.28 | 31.75±4.97 | 39.50±4.97 |
|  | Sarcophagidae | HGK60 | 0.00±0.00 | 10.50±1.5 | 20.75±4.82 | 22.75±2.49 | 0.00±0.00 | 9.50±1.50 | 15.00±4.95 | 22.00±3.24 |
|  |  | Z58 | 0.00±0.00 | 12.25±5.21 | 18.00±2.12 | 23.50±1.80 | 0.00±0.00 | 7.75±2.28 | 18.00±4.30 | 20.25±4.44 |
| Homoptera | Delphacidae | HGK60 | 17.25±1.78 | 21.00±3.24 | 31.75±2.59 | 25.00±3.08 | 17.75±2.17 | 22.75±2.59 | 30.25±3.70 | 29.75±1.92 |
|  |  | Z58 | 19.00±1.58 | 23.50±3.35 | 34.50±3.84 | 25.75±3.34 | 19.25±2.77 | 23.50±3.20 | 27.50±5.22 | 31.00±3.39 |
|  | Cicadellidae | HGK60 | 8.75±1.47 | 17.75±2.17 | 28.50±3.5 | 21.25±2.86 | 9.75±1.30 | 20.00±0.71 | 31.00±2.74 | 18.50±3.84 |
|  |  | Z58 | 10.25±1.47 | 17.50±2.69 | 29.75±2.86 | 22.25±2.17 | 11.00±1.87 | 20.25±1.92 | 32.50±3.91 | 19.50±3.50 |
|  | Aphidoidea | HGK60 | 15.75±3.76 | 27.50±3.35 | 36.50±3.91 | 28.00±2.35 | 17.75±2.59 | 27.00±3.94 | 29.00±4.95 | 31.75±2.38 |
|  |  | Z58 | 19.50±3.90 | 26.50±5.85 | 37.50±5.68 | 27.00±5.52 | 19.75±0.83 | 30.00±2.55 | 33.00±3.39 | 33.25±2.68 |
|  | Psyllida | HGK60 | 6.50±1.50 | 7.50±1.66 | 10.50±1.5 | 14.50±2.29 | 9.75±0.83 | 7.75±2.38 | 10.25±1.64 | 14.50±2.29 |
|  |  | Z58 | 7.75±2.86 | 7.75±2.86 | 11.75±2.28 | 12.25±2.38 | 11.50±2.5 | 8.00±2.55 | 11.25±2.59 | 14.50±2.50 |
| Thysanoptera | Thripoidea | HGK60 | 37.50±4.50 | 50.25±3.70 | 47.25±5.58 | 47.75±5.49 | 41.00±3.67 | 53.25±2.49 | 67.50±1.66 | 47.75±5.97 |
|  |  | Z58 | 44.25±3.76 | 49.00±6.16 | 46.50±3.20 | 42.25±3.27 | 41.75±4.26 | 54.75±1.79 | 66.75±3.03 | 49.00±5.79 |
| Neuroptera | Chrysopidae | HGK60 | 17.75±2.58 | 23.50±3.35 | 37.50±2.06 | 25.50±2.87 | 18.75±1.64 | 23.50±3.04 | 31.25±2.17 | 36.25±3.03 |
|  |  | Z58 | 17.25±3.56 | 23.25±3.35 | 17.25±3.56 | 24.75±2.49 | 18.25±2.05 | 24.75±3.56 | 35.00±3.54 | 32.25±3.34 |
| Hymenoptera | Ichneumonidae | HGK60 | 2.25±0.82 | 3.75±1.30 | 7.50±2.60 | 12.25±1.79 | 3.00±0.71 | 4.75±1.79 | 9.25±2.68 | 13.75±2.49 |
|  |  | Z58 | 1.50±0.50 | 4.25±1.48 | 8.00±2.45 | 14.25±2.86 | 3.50±0.87 | 7.00±2.24 | 9.75±2.28 | 12.25±2.28 |
|  | Trichogrammatidae | HGK60 | 3.25±0.82 | 4.50±1.12 | 8.50±3.64 | 11.25±1.48 | 2.75±0.83 | 4.00±1.87 | 10.00±2.55 | 9.75±1.92 |
|  |  | Z58 | 2.25±1.08 | 4.50±1.12 | 8.75±2.59 | 12.50±2.50 | 3.00±1.41 | 6.00±2.24 | 10.50±1.65 | 11.50±2.87 |
|  | Encyrtidae | HGK60 | 4.25±2.58 | 6.25±1.30 | 11.25±1.92 | 13.75±1.09 | 4.25±1.48 | 7.00±1.22 | 10.25±2.28 | 12.75±2.77 |
|  |  | Z58 | 2.50±1.50 | 6.25±1.64 | 10.25±3.56 | 14.25±2.28 | 4.50±2.30 | 9.00±1.87 | 8.75±2.86 | 13.50±1.12 |
|  | Chrysididae | HGK60 | 9.50±1.11 | 11.75±1.48 | 17.25±4.32 | 21.25±3.70 | 8.25±1.48 | 10.75±0.83 | 15.25±4.44 | 22.00±4.06 |
|  |  | Z58 | 8.00±1.87 | 11.25±2.49 | 17.75±4.15 | 25.50±2.96 | 8.00±1.87 | 11.75±2.59 | 15.75±3.49 | 20.75±4.32 |
|  | Sphecidae | HGK60 | 15.25±1.92 | 16.50±1.12 | 20.00±3.16 | 22.50±2.96 | 10.50±2.29 | 13.50±2.96 | 17.75±1.48 | 23.75±3.83 |
|  |  | Z58 | 13.25±2.38 | 15.25±2.17 | 19.00±2.74 | 22.50±2.29 | 10.00±1.22 | 14.00±2.00 | 16.50±3.35 | 23.25±3.27 |
|  | Pteromalidae | HGK60 | 5.25±1.47 | 7.25±1.48 | 12.00±2.12 | 15.00±2.12 | 3.75±1.48 | 7.25±1.48 | 12.25±3.49 | 16.00±3.67 |
|  |  | Z58 | 4.50±1.65 | 7.75±1.30 | 12.50±3.04 | 18.00±2.55 | 4.50±0.50 | 9.00±2.12 | 11.75±3.96 | 17.25±2.86 |
|  | Mymaridae | HGK60 | 3.50±1.11 | 7.00±1.58 | 11.25±3.11 | 15.50±2.18 | 2.75±1.48 | 10.00±1.58 | 14.00±3.16 | 16.75±3.27 |
|  |  | Z58 | 2.25±1.63 | 9.00±2.55 | 12.00±3.94 | 15.50±2.29 | 2.25±0.83 | 11.25±2.59 | 12.25±3.56 | 16.50±3.64 |
|  | Braconidae | HGK60 | 10.50±2.95 | 14.00±3.16 | 19.25±3.27 | 22.50±1.12 | 8.25±1.48 | 11.25±2.59 | 16.50±3.84 | 22.50±3.64 |
|  |  | Z58 | 9.00±1.87 | 13.75±3.27 | 18.50±2.96 | 21.50±0.87 | 9.00±1.58 | 12.50±2.96 | 15.00±1.87 | 20.50±3.64 |
| Coleoptera | Carabidae | HGK60 | 2.25±0.43 | 3.00±1.58 | 12.25±2.38 | 17.50±2.29 | 3.00±1.59 | 2.50±1.12 | 8.75±0.83 | 18.75±1.92 |
|  |  | Z58 | 3.00±0.71 | 3.75±1.48 | 11.25±2.77 | 18.50±2.5 | 2.50±1.12 | 4.25±1.48 | 9.50±1.50 | 17.75±2.28 |
|  | Coccinellidae | HGK60 | 8.75±2.16 | 16.50±2.69 | 28.25±3.11 | 27.25±1.92 | 9.50±1.11 | 13.75±2.69 | 23.25±3.27 | 22.75±2.86 |
|  |  | Z58 | 7.50±2.29 | 16.50±2.29 | 29.25±3.56 | 29.75±3.11 | 8.75±3.34 | 14.75±2.86 | 26.25±2.28 | 20.50±3.64 |
|  | Staphylinidae | HGK60 | 3.25±1.47 | 10.50±2.69 | 15.50±3.28 | 18.50±2.06 | 6.00±0.71 | 9.25±2.17 | 15.25±2.17 | 19.50±3.20 |
|  |  | Z58 | 3.50±2.17 | 9.50±2.06 | 14.50±3.64 | 18.25±3.42 | 5.50±2.062 | 10.25±2.95 | 16.25±3.27 | 18.25±3.42 |
| Orthoptera | Gryllidae | HGK60 | 9.00±1.58 | 12.25±2.17 | 27.00±4.47 | 32.75±2.86 | 10.50±1.66 | 12.25±1.48 | 23.00±3.54 | 34.25±1.09 |
|  |  | Z58 | 8.75±1.78 | 11.50±2.60 | 25.75±6.61 | 30.75±2.86 | 10.25±1.30 | 12.00±2.12 | 23.75±2.78 | 36.25±3.11 |
|  | Acrididae | HGK60 | 9.00±1.87 | 12.25±2.38 | 26.00±5.15 | 32.50±3.04 | 11.50±1.8 | 13.75±1.64 | 24.00±3.16 | 34.75±4.32 |
|  |  | Z58 | 9.25±1.92 | 12.25±3.34 | 27.25±4.44 | 31.00±2.74 | 11.75±1.48 | 13.75±3.49 | 26.00±3.39 | 35.75±4.60 |
|  | Tetrigoidea | HGK60 | 8.75±2.16 | 10.75±1.30 | 22.00±4.64 | 29.00±3.16 | 9.25±1.48 | 13.00±2.55 | 16.50±1.12 | 26.75±2.95 |
|  |  | Z58 | 7.75±1.29 | 10.50±2.60 | 23.00±3.40 | 28.00±5.61 | 10.50±1.5 | 12.00±2.12 | 18.00±2.55 | 28.75±3.35 |
| Araneae | Araneidae | HGK60 | 14.00±2.12 | 18.25±2.38 | 26.00±3.74 | 31.25±4.02 | 12.00±2.12 | 16.50±2.29 | 24.50±5.12 | 38.50±3.35 |
|  |  | Z58 | 16.75±2.68 | 19.25±1.48 | 26.00±3.74 | 32.25±2.17 | 11.00±3.16 | 18.25±1.92 | 27.25±4.32 | 34.75±5.31 |
|  | Thomisidae | HGK60 | 8.75±1.47 | 10.75±1.79 | 15.50±3.04 | 22.00±2.24 | 7.50±1.12 | 9.00±1.22 | 18.50±3.50 | 21.50±2.96 |
|  |  | Z58 | 9.00±2.12 | 10.25±1.30 | 16.50±3.64 | 23.50±2.06 | 8.00±2.74 | 13.00±2.74 | 19.50±1.80 | 20.25±4.15 |
|  | Salticidae | HGK60 | 8.25±2.27 | 11.25±2.28 | 17.25±4.82 | 22.25±4.82 | 6.25±2.49 | 10.50±2.06 | 19.00±3.24 | 24.50±3.20 |
|  |  | Z58 | 8.75±2.48 | 10.75±2.17 | 18.25±7.95 | 25.75±4.97 | 5.75±1.48 | 13.00±1.58 | 21.00±5.43 | 23.00±7.18 |
| Odonata | Coenagrionidae | HGK60 | 9.00±1.58 | 11.75±1.92 | 17.00±1.87 | 22.25±1.92 | 7.75±2.49 | 9.50±2.06 | 17.00±1.87 | 22.25±2.95 |
|  |  | Z58 | 10.00±2.73 | 12.00±1.87 | 19.25±6.53 | 23.50±5.59 | 7.00±1.58 | 10.75±1.79 | 19.25±6.53 | 23.00±5.24 |
